# Folated Synperonic-Cholesteryl Hemisuccinate Polymeric Micelles for the Targeted Delivery of Docetaxel in Melanoma

**DOI:** 10.1155/2015/746093

**Published:** 2015-03-08

**Authors:** Jaleh Varshosaz, Somayeh Taymouri, Farshid Hassanzadeh, Shaghayegh Haghjooy Javanmard, Mahboobeh Rostami

**Affiliations:** ^1^Department of Pharmaceutics, School of Pharmacy and Novel Drug Delivery Systems Research Centre, Isfahan University of Medical Sciences, Isfahan 81746 73461, Iran; ^2^Department of Medicinal Chemistry, School of Pharmacy, Isfahan University of Medical Sciences, Isfahan 81746 73461, Iran; ^3^Applied Physiology Research Center, Isfahan University of Medical Sciences, Isfahan 81746 73461, Iran

## Abstract

The objective of this study was the synthesis of folic acid- (FA-) targeted polymeric micelles of Synperonic PE/F 127-cholesteryl hemisuccinate (PF127-Chol) for specific delivery of docetaxel (DTX). Targeted or nontargeted micelles loaded with DTX were prepared via dialysis method. The effects of processing variables on the physicochemical properties of targeted micelles were evaluated using a full factorial design. After the optimization of the polymer/drug ratio, the organic solvent type used for the preparation of the micelles, and the temperature of dialyzing medium, the* in vitro *cytotoxicity and cellular uptake of the optimized micelles were studied on B16F10 melanoma cells by flow cytometry and fluorescent microscopy. The anticancer efficacy of DTX-loaded FA-PF127-Chol was evaluated in mice bearing melanoma tumor. Optimized targeted micelles had the particle size of 171.3 nm, zeta potential of −7.8 mV, PDI of 0.325, and a high encapsulation efficiency that released the drug within 144 h. The MTT assay indicated that targeted micelles carrying DTX were significantly more cytotoxic, had higher cellular uptake, and reduced the tumor volume significantly more than the nontargeted micelles and the free drug. FA-PF127-Chol could be, therefore, a promising biomaterial for tumors overexpressing folate receptors.

## 1. Introduction

Melanoma is the most leading cause of skin cancer mortality, accounting for 5% of all new cancers reported in the United States during 2012 [1]. The incidence of malignant melanoma is increasing annually at a rate faster than that of other types of cancers [2]. In US, 8700 deaths occurred due to melanoma in 2012 and 9840 new deaths are estimated in 2014, thereby testifying the increase in the incidence rate of this disease [3]. Melanoma of skin can happen at any age, but most cases are affected within the age range of 55 to 64. Furthermore, white people are more susceptible to melanoma than other races. Melanoma is curable if detected early with an overall 5-year survival rate of 95%, but it suffers from poor diagnosis with an overall 5-year survival rate of 61.7% and 15.7% for regional and distant metastasis, respectively [1]. Thus, there is an urgent need for the early detection and treatment of melanoma. Current treatments of melanoma are surgery, radiation therapy, and systemic therapy with cytotoxic or immunotherapeutic agents alone or in combination [4]. Although surgery and radiation therapy can be effective in the early phase of melanoma, systemic therapy with cytotoxic drugs is the main treatment of metastatic melanoma [5].

Docetaxel (DTX) is a member of taxane group drugs extracted from the European yew tree with perfect effectiveness against various cancers including melanoma, lung, ovary, breast, and leukemia [6]. Due to its low water solubility, it is formulated using polysorbate 80 and ethanol as cosolvents. However, DTX application in clinic is limited due to several adverse effects caused by solvent system and the nonspecific distribution of drug in the healthy organs.

Nanotechnology provides a promising solution in the treatment and diagnosis of cancer. Passive targeting of nanoparticles in tumor tissue is possible due to the inherent nature of tumor tissue, which results from its rapid growth and angiogenesis. The leaky nature of angiogenic vessels with gap sizes in the range of nanometers to few microns [7] and the poor lymphatic drainage result in the enhanced permeation and retention (EPR) of nanoparticles and large molecules in the tumor tissue [8]. An appropriate drug delivery system should not only facilitate drug accumulation in the tumor tissue, but also increase drug concentration inside the tumor cells [9]. Tumor stroma and high fluid pressure in the center of tumor tissue are barriers that limit the penetration of nanoparticles in the tumor tissue [7]. To solve this problem and increase the cellular accumulation of nanoparticles, specific drug delivery systems have been developed using various targeting ligands including peptides [10, 11], carbohydrates [12], aptamers [13], vitamins [14, 15], and monoclonal antibodies [16]. The use of the active targeting system provides more opportunities for the selective and quantitative drug delivery to the tumor site. Among these, folic acid (FA) has become a popular targeting moiety due to the overexpression of folate receptors (FR) on various types of human cancers, high binding affinity of FA (*k*_*d*_ = 10^−10^ M) to FR, and the role of FA in cellular mediated endocytosis [15, 17]. Availability, low cost, stability, and nonimmunogenicity are other advantages that have enhanced the attractiveness of FA as the targeting agent [15, 17, 18]. Polymeric micelles are self-assembled nanoparticles with a core-shell structure [19]. In many researches, especially in the area of anticancer drug delivery, polymeric micelles have been used as a carrier due to several unique advantages such as small size, which facilitates drug accumulation in the tumor tissue via EPR effect, their potential for the solubilization of hydrophobic drugs, and the sustained release behavior. In addition, they have been shown to prolong drug circulation time via preventing renal clearance and reticuloendothelial system (RES) removal [20, 21]. To design suitable polymeric micelles as a drug delivery system, polymer selection is critical. Synperonic copolymers are synthetic amphiphilic copolymers arranged in the triblock structure (PEO)_*x*_-(PPO)_*y*_-(PEO)_*x*_ [22]. Synperonic copolymers are widely applied in pharmaceutics because of their suitable features [23]. They are FDA-approved, biocompatible, and not so cytotoxic and immunogenic [24]. In addition, they could be a biological response modifier inhibiting drug efflux transport proteins such as P-glycoprotein, which contributes to increasing the sensitivity of multidrug resistance tissue to anticancer drugs [25, 26]. They have also been used to increase drug solubility and bioavailability [27]. However, Synperonic copolymers have high critical micelle concentration (CMC) and are susceptible to micelle dissociation upon dilution, so structural modification with lipophilic segments or preparation of mixed micelles could be regarded as a promising approach that can be employed for solving this problem [28, 29]. For example, stearic acid was linked to PF127 to enhance the stability of the micelles. Stearic acid conjugation led to a remarkable decrease in the CMC of PF127 [29]. In another study, Varshosaz et al. [30] introduced a micellar system composed of Synperonic PE/F 127 and cholesteryl hemisuccinate (PF127-Chol). Cholesteryl hemisuccinate was selected to modify Synperonic PE/F 127 structure because of several reasons including biocompatibility and a highly hydrophobic sterol structure, which both increase drug loading due to the compatibility of hydrophobic drug and cholesterol bearing material [31–33]. In addition, cholesteryl hemisuccinate has an anticancer effect due to its ability to inhibit enzymes of DNA polymerase and DNA topoisomerase, thereby contributing to DNA replication and repair and cell division [34]. The CMC value of PF127-Chol copolymers [30] was found to be 41 *µ*g/mL, which was significantly lower than the CMC value of Synperonic micelles (1–25 mg/mL) [35, 36] and low molecular weight surfactants such as sodium dodecyl sulfate (2.3 mg/mL) [37], thereby confirming more stability of PF127-Chol micelles against dilution in aqueous solution. The results were encouraging, but this system was lacking a targeting moiety to direct the drug to the tumor tissue. Many researchers have employed active targeting for the enhanced delivery and maintenance of therapeutic agents in the tumor tissue. In a study conducted by Zhang et al. [9], the use of curcumin-loaded folate modified self-microemulsifying drug delivery system (FSMEDDS) showed superior cytotoxic activity compared to the nontargeted form of this carrier system in both HT29 and HeLa cell lines. In another approach, Zhou et al. [38] developed folate targeted chitosan-gemcitabine (PEG-FA-Gem-Chi) core-shell nanoparticles targeted to the pancreatic cancer. The* in vitro* results showed that FA targeting moiety increased the cytotoxicity of the nanoparticles in COLO357 cells. The* in vivo* study in nude mice bearing human pancreatic cancer showed that PEG-FA-Gem-Chi had a stronger antitumor activity compared to Gem and PEG-Gem-Chi.

In the present study, FA-grafted Synperonic PE/F 127-cholesteryl hemisuccinate copolymer (FA-PF127-Chol) was synthesized and used to develop a carrier for the delivery of DTX. DTX-loaded polymeric micelles were prepared via the dialysis method. The physicochemical characteristics of nanomicelles including particle size, zeta potential, morphology, loading efficiency, and* in vitro* drug release were investigated. B16F10 melanoma cell line, which has been demonstrated to overexpress FR, was used as the tumor model of melanoma, and the anticancer effect and the cellular uptake of DTX-loaded polymeric micelles were evaluated. Then these cells were used for inducing tumor in C57BL6 mice and the antitumor effect of the designed micelles was compared to that of the free drug.

## 2. Experimental

### 2.1. Materials

DTX was obtained from Cipla (India). Synperonic PE/F 127 (PF127), cholesterol (Chol), succinic anhydride, anhydrous dimethyl sulfoxide (DMSO), folic acid (FA), 1,1′-carbonyldiimidazole (CDI), 4-dimethyl amino pyridine (DMAP), dicyclohexylcarbodiimide (DCC), dichloromethane (DCM), acetonitrile, and methanol were purchased from Merck Chemical Company. Dialysis bag (cut-off: 6000–8000 Da, 2000 Da), coumarin 6 (C6), and 3-[4,5-dimethylthiazol-2-yl]-2,5-diphenyltetrazolium bromide (MTT) were obtained from Sigma Company (USA). Trypsin, fetal bovine serum (FBS), phosphate buffer saline (PBS), Roswell Park Memorial Institute-1640 medium (RPMI-1640 medium), Dulbecco's Modified Eagle Medium (DMEM), penicillin, and streptomycin were sourced by Gibco Laboratories (USA).

### 2.2. Cell Lines

B16F10 melanoma cell line, HepG2 human liver hepatocellular carcinoma cell line, and mouse fibroblast cells L929 were provided from Pasteur Institute (Iran). B16F10 and HepG2 cells were grown in RPMI-1640 and L929 cells were cultured in DMEM supplemented with 10% (v/v) FBS, 100 IU/mL penicillin, and 100 *μ*g/mL streptomycin sulfate. The cultures were maintained in humidified atmosphere containing 5% CO_2_ at 37°C.

### 2.3. Synthesis of FA-Grafted Synperonic PE/F 127- (PF127-) Cholesteryl Hemisuccinate

Briefly, 0.616 mmol of cholesteryl hemisuccinate (or 0.300 g) was dissolved in anhydrous DCM. 0.616 mmol of DMAP (or 0.0752 g) and 0.616 mmol of DCC (or 0.127 g) were added. After the completion of the reaction (24 h at room temperature under nitrogen atmosphere), dicyclourea was removed via filtration and the filtrate was added dropwise to the solution of Synperonic PE/F 127 (0.616 mmol, 7.7 g) in the anhydrous DCM. The reaction was stirred under nitrogen atmosphere for 24 h at room temperature. For the conjugation of FA to PF127-Chol, 0.2 mmol of FA (or 88.28 mg) was dissolved in the anhydrous DMSO and after complete dissolution 0.22 mmol (35.2 mg) of CDI was added. The activation reaction was performed at room temperature under nitrogen atmosphere in darkness for 24 h. After that, the solution of 0.05 mmol of PF127-Chol (or 649.33 mg) in DMSO was added to activated FA. The reaction was allowed to continue under nitrogen atmosphere in darkness for further 24 h. The mixture was then dialyzed against deionized water for 48 h using dialysis bag (2000 Da) to remove the unreacted reagents. Finally, the product was obtained via lyophilization. The chemical structure of the synthesized copolymer was evaluated by proton nuclear magnetic resonance (^1^HNMR) and Fourier transform infrared (FTIR). HNMR spectra were recorded on Bruker BioSpin AC-80 400 MHz ^1^HNMR spectrophotometer (Germany) using d^6^-DMSO as the solvent and FTIR spectra were obtained by FTIR spectrophotometer (Rayleigh, WQF-510/520, China) using KBr pellets. To provide evidence for the chemical reaction and the purification of the product of reaction, dialysis was continued for further 7 days. The samples were collected at time intervals of 24, 48, and 72 h and 7 days and their chemical structures were evaluated using ^1^HNMR.

### 2.4. Preparation of DTX-Loaded Polymeric Micelles

**Figure 1:**
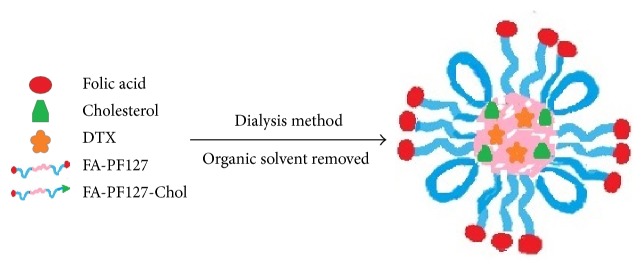
Schematic representation of structure of micelles.

After the synthesis of FA-grafted PF127-Chol copolymer, DTX-loaded micelles were prepared by the dialysis method. For this purpose, a constant amount of DTX and different amounts of copolymer (Table 1) were dissolved in DMSO or DMF by the sonication method. To produce polymeric micelles, the solution of DTX and the copolymer was dialyzed against distilled water in various temperatures (25 or 40°C) for 24 h using the dialysis bag (cut-off: 6000–8000) to remove the organic solvent and the free drug. The medium was changed every 2 h until 8 h. Then the obtained micelle dispersion was freeze dried for further studies. The schematic representation of the structure of micelles is shown in Figure 1.

### 2.5. Experimental Design and Analysis

To find the optimal conditions for producing nanoparticles by taking the appropriate output responses including particle size, zeta potential, encapsulation efficiency, and release efficiency, the Design-Expert software (ver. 7.2, USA) was used. Three different processing variables including polymer/drug ratio, organic solvent, and dialysis temperature were studied, each in two levels. Eight different formulations were designed by a general full factorial design (Table 1). All experiments were done in triplicate. The optimum conditions were determined by an optimization process to yield a heightened performance.

### 2.6. Determination of DTX Encapsulation Efficiency

Encapsulation efficiency was determined by dissolving 1 mg of freeze-dried product in a mixture of 50 : 50 acetonitrile/water. The obtained solution was filtered via 0.45 *µ*m filter and DTX concentration was determined using a Waters HPLC system (Waters, USA) equipped with 515 HPLC pump, dual *λ* absorbance detector, and RP-C18 column (Fortis, 250 mm × 4.6 mm, 5 *µ*m). The mobile phase consisted of a mixture of acetonitrile/water (65 : 35) at the flow rate of 1 mL/min. The calibration curve was recorded from 0.250 to 40 *µ*g/mL in acetonitrile. Injection volume was about 40 *µ*L. The UV detector was set at the detection wave length of 230 nm. Drug loading efficiency was determined as the determined amount of drug in 1 mg of freeze-dried sample per initial amount of drug used in every mg of the sample.

### 2.7. Scanning Electron Microscopy (SEM) and Transmission Electron Microscopy (TEM)

The morphological study of DTX-loaded optimized FA-PF127-Chol micelles was carried out using SEM (FE-SEM; HITACHI S-4160, Japan) and TEM (Philips EM 208S transmittance electron microscopy). For SEM study, a small amount of freeze-dried micelles was coated with gold under vacuum using a sputter coater. Then the shape of micelles was examined by SEM. For TEM study, some droplets of a well-dispersed sample of optimized formulation were placed on carbon-coated copper grid and then air-dried at room temperature. Then the images were taken using TEM.

### 2.8. Particle Size, Polydispersity Index (PDI), and Zeta Potential of the Micelles

Particle size, PDI, and zeta potential of the blank and drug-loaded micelles of FA-PF127-Chol and PF127-Chol were determined by dynamic light scattering method using Malvern nanosizer. 1 mg of lyophilized micelles was dispersed in deionized water and analyzed at the scattering angle of 90° at room temperature.

### 2.9. Stability Studies of the Micelles

To study the storage stability, the lyophilized forms of DTX-loaded micelles were stored at room temperature for 2 months. The samples were taken to determine the particle size, zeta potential, PDI, and drug content of micelles at the beginning and 2 months after preparation.

To study the stability of the micelles against dilution, the optimal formulation of FA-PF127-Chol was diluted with PBS solution in different dilutions and stored at room temperature for 24 h. Then, the particle size and PDI of the micelles were determined.

### 2.10. *In Vitro* Release of DTX

*In vitro* release of DTX from nanomicelles was evaluated by the dialysis method. An appropriate amount of freeze-dried sample was dispersed in phosphate buffer solution (PBS) at the pH of 7.4 and placed in the dialysis bag (cut-off 6000–8000 Da). The bags were immersed in an appropriate amount of PBS at the pH of 7.4, which contained 0.5% Tween 80. At predetermined time intervals, an appropriate amount of medium was removed and replaced with fresh medium. The amount of released drug was determined by RP-HPLC as described earlier. The parameter of release efficiency was obtained after 144 h to compare the release behavior of DTX from the micelles.

Release efficiency until 6 days (or 144 h) (RE_144_%) of release test was calculated by

(1) $$\begin{array}{c} {\text{RE}}_{144}\%=\frac{\int_{0}^{t}y\cdot dt}{y_{100}\cdot t}\times 100, \end{array}$$

where *y* is the released percent at time *t*.

### 2.11. *In Vitro* Cytotoxicity Assays

The cytotoxicity of free DTX, bare and DTX-loaded PF127-Chol, and optimized FA-PF127-Chol micelles (P12S25) was evaluated on hepatocellular carcinoma HepG2 cells (folate receptor negative) and B16F10 melanoma cells (folate receptor positive) by MTT assay. DTX-loaded nontargeted PF127-Chol micelles were prepared by a similar method with the same polymer/drug ratio of the optimized targeted micelles. Free DTX solution was prepared by dissolving an appropriate amount of drug in DMSO 1%. Some RPMI-1640 containing 10% of fetal bovine serum and 1% of penicillin-streptomycin was used as the medium for cell cultivation. Cells were incubated in an atmosphere containing 5% CO_2_ at 37°C. B16F10 and HepG2 cells were seeded in 96-well plates at the density of 5 × 10^3^ and incubated for 24 h to allow cell attachment. Then, the cells were exposed to different concentrations of free DTX, DTX-loaded PF127-Chol, and optimized FA-PF127-Chol micelles (P12S25) for 48 h. At the end of the incubation time, 20 *µ*L of MTT solution at 5 mg/mL concentration was added to each well and incubated for further 3 h. Then, the medium was discarded and 150 *µ*L of DMSO was added to each well to dissolve formazan crystals. The toxicity of the polymeric micelle carriers, both nontargeted and targeted micelles, was tested as described above in the equivalent concentration of materials used in drug-loaded micelles. The absorbance of each well was measured at 570 nm using a microplate reader. The medium was used as the blank. The number of viable cells was determined using the following equation:

(2) Cell  viability%=Mean  absorbance  of  sampleCell  viability%h=M− mean  absorbance  of  blankCell  viability%=·Mean  absorbance  of  controlCell  viability%hhl=M− mean  absorbance  of  blank−1Cell  viability%=×100.

The cytotoxicity of bare PF127-Chol and FA-PF127-Chol micelles was also evaluated on mouse fibroblast L292 cells. L292 cells were grown in DMEM supplemented with 10% of fetal bovine serum and 1% of penicillin-streptomycin. L292 cells were seeded in 96-well plates at the density of 5 × 10^3^ and incubated for 24 h to allow cell attachment. Then, L292 cells were exposed to different concentrations of blank PF127-Chol and FA-PF127-Chol micelles for 24 and 48 h and cell viability was measured by MTT method.

### 2.12. *In Vitro* Cellular Uptake Study

The cellular uptake of PF127-Chol or FA-PF127-Chol micelles was studied using a fluorescent microscope (CETI, Belgium) and flow cytometry device (BD FACSCalibur, US). C6-loaded polymeric micelles were prepared by the dialysis method. For this experiment, 1 mg of C6 and 120 mg of PF127-Chol copolymer or FA-PF127-Chol were dissolved in DMSO and dialyzed against 1 liter of deionized water for 24 h using the dialysis bag (cut-off 6000–8000 Da). After that, the dispersion was collected and freeze dried. For flow cytometry study, B16F10 cells were seeded at the density of 2 × 10^5^ in 24-well plates and incubated at 37°C for 24 h. After that, the cells were incubated with free C6 or C6-loaded PF127-Chol micelles or C6-loaded FA-PF127-Chol for 3 h. After 3 h, the cells were washed with PBS to remove the micelles not internalized in cells and 150 *µ*L of trypsin was added (for 3 min). The medium containing 10% of FBS was then added to stop trypsin action. Finally, the cells were collected by centrifugation at 1800 rpm for 3 min, resuspended in 1 mL of PBS (pH 7.4), and analyzed by flow cytometer. For the fluorescent microscopy study, B16F10 and HepG2 cells were seeded at the density of 2 × 10^5^ in 24-well plates and incubated at 37°C for 24 h. After 3 h incubation of cells with free C6, C6-loaded PF127-Chol micelles, or C6-loaded FA-PF127-Chol, the medium was discarded and the cells were washed with 200 *µ*L of PBS. Then, the cell monolayer was imaged with a fluorescent microscope.

### 2.13. *In Vivo *Studies

#### 2.13.1. Animals

Male C57BL6 mice aging 4–6 weeks (20–30 gr) (Pasteur Institute, Iran) were used to carry out the antitumor efficacy study. The mice were housed at 25°C and 55% humidity in a light controlled house with free access to food and water. All animal procedures were carried out in compliance with the protocol established by the animal study committee of Isfahan University of Medical Sciences.

#### 2.13.2. The* In Vivo *Antitumor Efficacy

Antitumor activity of various formulations was evaluated in C57BL6 mice bearing melanoma. The mice were subcutaneously injected with a 200 *µ*L of B16F10 melanoma cells suspension containing 2 × 10^6^ cells on the right flank. When tumor volume reached about 100–200 mm^3^ (measured on day 0), the mice were divided into four groups (*n* = 5) and treated intravenously via tail vein with Taxotere (10 mg/kg), DTX-loaded FA-PF127-Chol (10 mg/kg), DTX-loaded PF127-Chol (10 mg/kg), and normal saline 0.9% every 3 days with the total of 3 doses. The tumor size and animal body were monitored every 3 days for 12 days after the first drug administration. Tumor volume was calculated by following equation:

(3) $$\begin{array}{c} \text{Tumor}\;\text{volume}=0.5\times b^{2}\times a, \end{array}$$

where *a* and *b* are the longest and shortest diameters of tumors, respectively. At day 15, mice were sacrificed and tumors were removed and weighted. Then the tumor tissue was fixed in formalin for 48 h, embedded in paraffin, and cut into 5 *µ*m slices. The sections were H&E stained and were examined under optical microscope (Olympus CX 21, Japan).

In another experiment, survival times were monitored from the first day of administration to the day of death. The survival data were presented as Kaplan-Meier plan.

## 3. Results and Discussion

### 3.1. Synthesis of FA-Grafted Synperonic PE/F 127-Cholesteryl Hemisuccinate Copolymer

In the present study, a novel, effective, economical, reliable, and safe method was developed for the modification of PF127. PF127-Chol copolymer was synthesized by coupling cholesteryl hemisuccinate with PF127 via an ester bond. We prepared cholesteryl hemisuccinate by the reaction of cholesterol with an excess amount of succinic anhydride. The synthesis was easy, rapid, and high yielding. After that, the carboxyl group of the cholesteryl hemisuccinate was activated with DCC and DMAP and then linked to the hydroxyl group of PF127.

**Figure 2:**
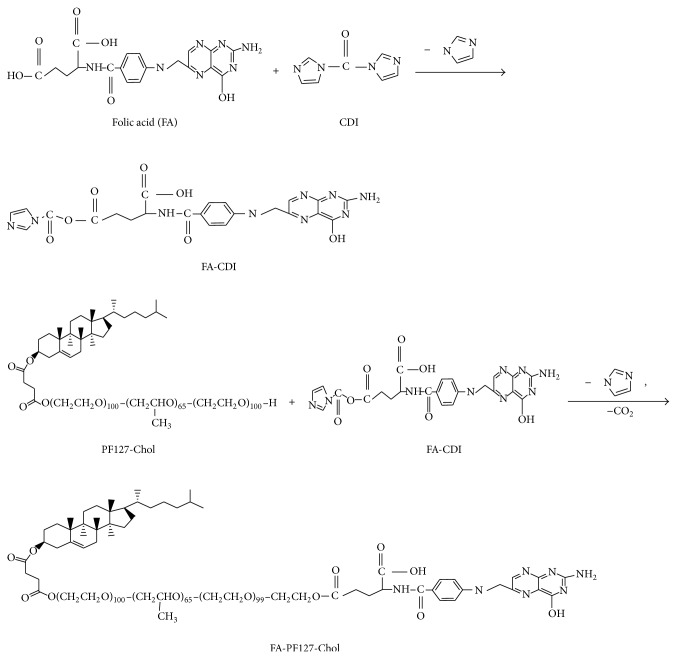
Schematic illustration of the synthesis procedure of FA-grafted PF127-Chol.

Figure 2 shows the synthesis procedure of FA-PF127-Chol. At first, FA was activated with CDI. Then, the activated FA was attached to PF127-Chol. Finally, the unconjugated FA was removed by dialysis against water using dialysis bag (cut-off 2000 Da).

**Figure 3:**
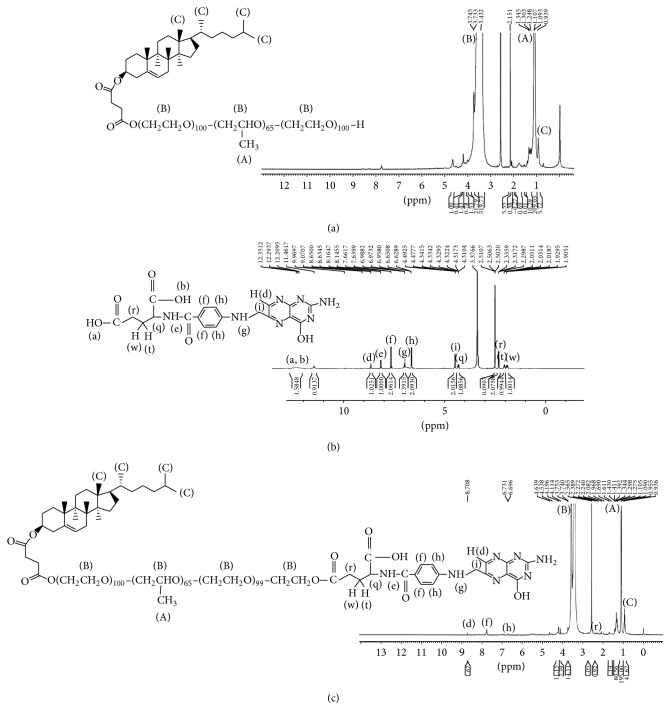
^1^HNMR spectra of (a) PF127-Chol, (b) pure FA, and (c) FA-PF127-Chol.

^1^HNMR and FTIR spectra of FA-PF127-Chol copolymer in d^6^-DMSO demonstrated the successful formation of the product. The ^1^HNMR spectra of PF127-Chol, pure FA, and FA-conjugated PF127-Chol in d^6^-DMSO are shown in Figure 3. As illustrated, the peaks at *σ* = 0.955 ppm were attributed to the methyl groups of cholesteryl hemisuccinate. The assigned peaks at *σ* = 1.09 ppm and *σ* = 3.2–3.7 ppm were related to CH3 group of polypropylene oxide (PPO) and CH2CH2O groups of PPO and polyethylene oxide (PEO) of PF127, respectively. The peaks at *σ* = 2.389 ppm, *σ* = 6.696 ppm, 7.758 ppm, and *σ* = 8.708 ppm were related to aliphatic, aromatic, and pteridine protons of FA, respectively. The ^1^HNMR spectra of pure FA showed that characteristic peak related to COOH at *σ* = 12.351 ppm was disappeared in ^1^HNMR of the final product. This could be due to ester bond formation between PF127-Chol copolymer and FA. The degree of the substitution of FA on each unit of PF127-Chol copolymer was calculated from ^1^HNMR spectrum of the final product. From the integration ratio of signals observed at *σ* = 8.708 ppm (pteridine proton of FA) and *σ* = 1.09 ppm (CH3 group of PPO), the FA mole ratio to the PF127-Chol was about 1.67 to 1. As described previously, the degree of the substitution of Chol on each PF127 unit was about 45%; then, overall, in 50% of PF127 chains, FA was attached to PF127 from both free OH groups on the two sides of Synperonic chain. In the remaining chains, the attachment was approximately 50 : 50 for both cholesterol and FA.

**Figure 4:**
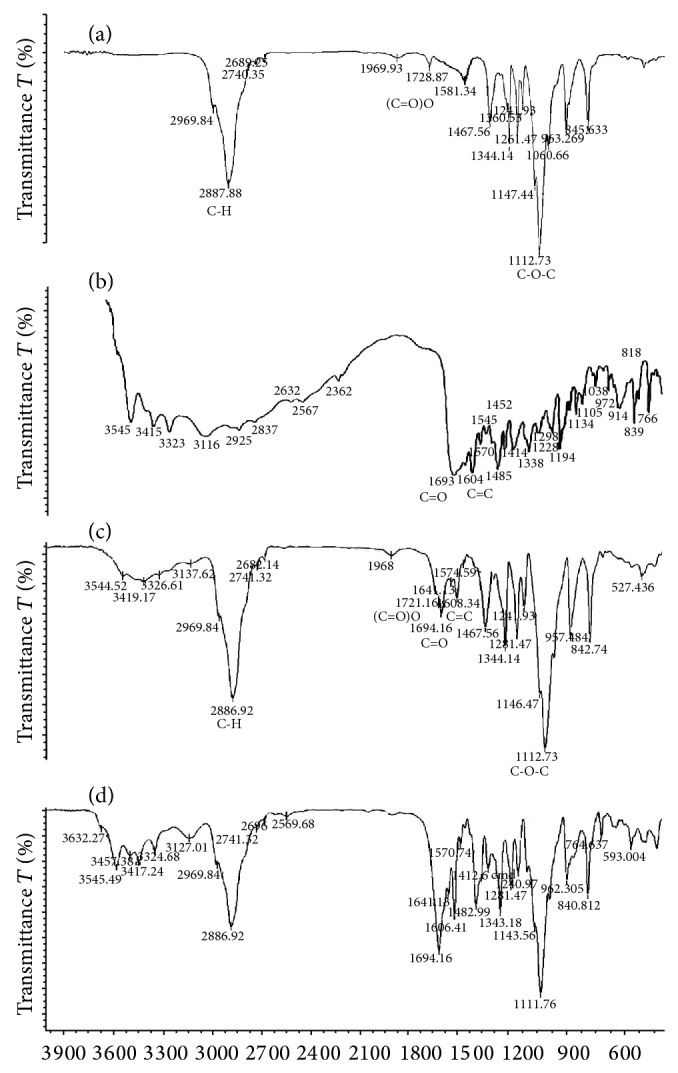
FTIR spectra of (a) PF127-Chol, (b) pure FA, (c) FA-PF127-Chol, and (d) physical mixture of FA and PF127-Chol.

Comparison of the integrated ratio of the selected signal of FA with PF127 showed that the constant ratio, after 7 days of dialysis, could be due to chemical conjugation rather than the physical mixture of the starting material. Conjugation of FA to PF127-Chol was also confirmed by FTIR analysis.  Figure 4 presents FTIR spectra of pure FA, PF127-Chol, and FA-PF127-Chol, respectively. FTIR spectrum of FA-PF127-Chol showed characteristic absorption bands at 2886.92 cm^−1^ and 1112.73 cm^−1^ that could be assigned to C–H and C–O–C stretching modes of PF127, respectively. The emergence of the assigned bands at 1608.34 cm^−1^ (C=C) and 1694 cm^−1^ (C=O) confirmed the presence of folic acid in the final product. In addition, the appearance of a new stretching band around 1721 cm^−1^ could reveal the formation of a new ester bond in the product. In comparison to FTIR spectrum of the physical mixture, the decrease in signal intensity around 3100–3600 cm^−1^ and 1694 cm^−1^ and the lack of absorption band at 1700 cm^−1^ or a higher area in the spectrum of the physical mixture suggested FA binding to PF127-Chol via ester bond.

### 3.2. Physical Characteristics of Micelles

The method used for the preparation of polymeric micelle depends on copolymer solubility. In the case of copolymers not readily dissolved in water and volatile organic solvents, dialysis method is the most common method used for the encapsulation of poorly water soluble drugs [37, 39]. In the present study, DTX-loaded polymeric micelles were prepared via dialysis method. The copolymer and the drug were dissolved in DMSO or DMF as an organic water miscible solvent and dialyzed against water. Drug-loaded self-assembled polymeric micelles were formed by the removal of organic solvent [40]. This method is easy and can produce a homogenous narrow size distribution of nanomicelles [37, 41]. Particle size is known as the most important characteristic of nanoparticles that can affect blood circulation time and tumor accumulation via EPR effect [6], efficiency, and pathway of the cellular uptake of NPs [42]. The pore cut-off size of porous blood vessels in tumor tissue was tumor-dependent between 0.2–2 *µ*m, but in the majority of tumors, it was approximately 380–780 nm [43]. Many studies have shown that the optimum size of nanoparticles to penetrate the cancerous tissue is below 400 nm, but to get long circulation time and escape from removal by reticuloendothelial system (RES), a particle size below 200 nm is necessary [44, 45].

Physicochemical properties of the blank micelles are shown in Table 2. The average particle size of the blank FA-PF127-Chol and PF127-Chol micelles was in the range of 86.25–192.3 nm. Except for P3F25, other formulations did not show any significant difference in size upon loading the DTX. The significant increase in the particle size of P3F25 formulation could be attributed to increasing the micellar core volume that resulted from DTX incorporation (solubilization) in the micelles core [46].

The effect of different formulation parameters such as polymer/drug ratio, solvent type, and temperature on the physicochemical properties of nanomicelles has been investigated and discussed in detail in our earlier article [47]. To optimize the production condition of the nanomicelles, the computer optimization process was done by Design-Expert software (version 7.2, US) and a desirability function determined the optimized levels of the independent variables of the process. All responses were fitted to the linear model. The constraint of particle size was 83.46–260.57 nm with targeting the particle size on minimum values; for zeta potential, it was from −4.72 to −15.6 mV while the target was set in the range of the obtained results. For the loading efficiency, the constraint was 65.39–100% with the goal set at the range of the obtained data (Table 3), and the RE_144_% constraint was 40–80% with the target set at the range of the obtained data in Table 3. For PDI, it was 0.24–0.78 while the target was set on minimum. By considering the data in Table 3, optimization was carried out by Design-Expert software with the desirability of 85% and the optimized situation for processing the micelles was suggested to be the formulation of P12S25, that is, using 5 mg of DTX, 60 mg of copolymer, and DMSO as the organic solvent and conducting dialysis at room temperature.

In our pervious study [47], we showed that the polymer/drug ratio was the most effective variable on particle size. With increasing the polymer concentration, the particle size was decreased. That could be attributed to the increase in micelles encapsulation efficiency and hydrophobicity, which resulted in the shrinkage of nanomicelles and the removal of water [48]. The mean particle size and PDI of both optimized DTX-loaded FA-PF127-Chol (formulation of P12S25) and DTX-loaded PF127-Chol micelles are represented in Table 3. The average PDI of the drug-loaded micelles was in the range of 0.2–0.78. The PDI of the optimized formulation (P12S25) was approximately 0.3, indicating that micelles had a relatively narrow size distribution. The low PDI for both types of optimized targeted and nontargeted polymeric micelles showed the narrow distribution width of micelles and the homogeneity of dispesion. The encapsulation efficiency of DTX in FA-PF127-Chol and PF127-Chol micelles has been summarized in Table 3. In both FA-PF127-Chol and PF127-Chol micelles, high encapsulation efficiency was obtained.

The zeta potential of the blank and DTX-loaded FA-PF127-Chol micelles ranged from −1.73 to −15.6 mV. It can be seen from Tables 2 and 3 that the loading of DTX into nanoparticles could affect zeta potential. A slight increase in the absolute value of zeta potential of blank micelles with drug loading or drug-loaded micelles by increasing the drug content (drug/polymer ratio) indicated that the drug was solubilized not only in the micelles core, but also in the micelles periphery.

For optimized FA-PF127-Chol (formulation of P12S25) and PF127-Chol micelles, zeta potential was −7.8 and −2.12, respectively. The results showed the increase in the absolute value of zeta potential (more negative) for FA-PF127-Chol micelles compared to PF127-Chol ones. The more negative charge of micelles could be due to the carboxylate group of FA located on the surface of the micelles. The other possible reason could be the smaller particle size of FA-PF127-Chol micelles compared to PF127-Chol. It has been shown that as particle size is decreased, the charge density on the surface of the micelle is increased too [37].

**Figure 5:**
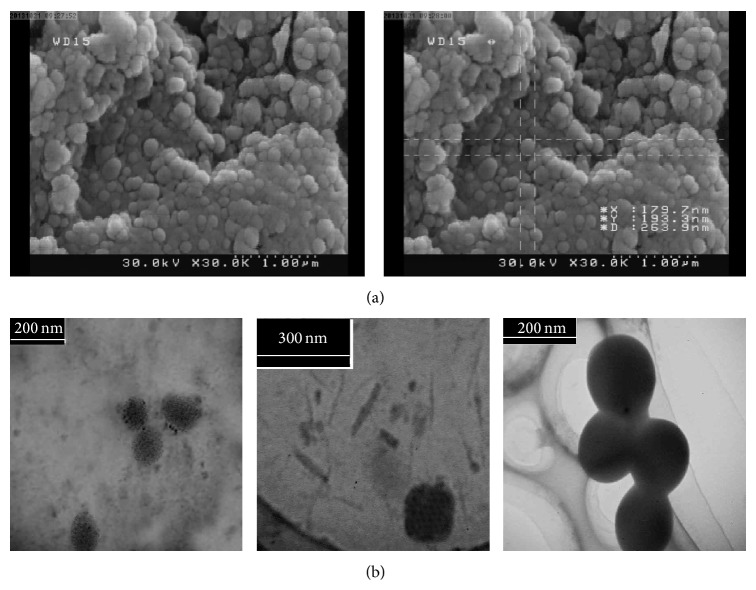
(a) SEM and (b) TEM images of DTX-loaded optimized FA-PF127-Chol micelles.

The morphology of nanomicelles was studied by SEM and TEM. SEM and TEM images (Figure 5) of DTX loaded in optimized FA-PF127-Chol micelles showed smooth spherical particles with a particle size nearly close to that obtained by the dynamic light scattering (DLS) method. However, the diameter of micelles observed by TEM and SEM was a little bit smaller than that of DLS method. DLS method determined the hydrodynamic diameter of micelles but TEM and SEM exhibited the dried state of the micelles. The larger particle size obtained from DLS method, compared to that of SEM and TEM images, might be attributed to shell hydration in aqueous media.

### 3.3. Stability Studies of the Micelles

Drug-loaded micelles were stored at 25°C for 2 months and storage stability was evaluated by monitoring the particle size, zeta potential, PDI, and drug content for 2 months after preparation (Table 4). In all formulations, drug content was not changed obviously during storage. A stable micellar system had to maintain the ratio of particle size in the range of 1 ± 0.3 compared to their initial size during storage [49]. Higher changes indicated the dissociation or aggregation of the micelles. It was observed that the formulations of P12F25, P3S40, and P12S40 showed some aggregation that resulted in their particle size enhancement and the decrease of their PDI. This could be due to the smaller original size of these formulations. Smaller nanoparticles had more surface free energy and greater aggregation tendency [50]. Other formulations showed high stability and their particle size change was in an acceptable range during storage.

**Figure 6:**
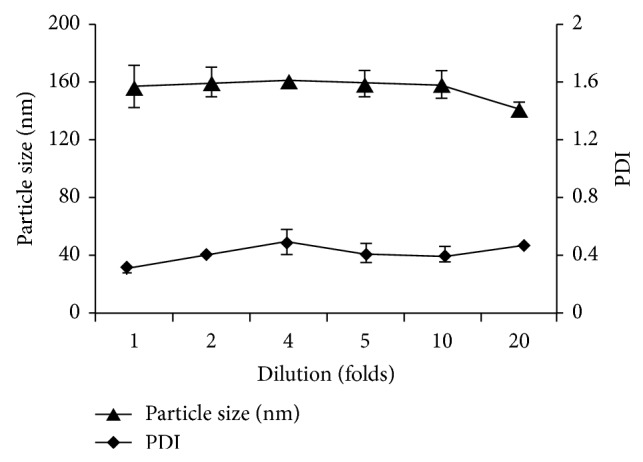
Particle size and size distribution of FA-PF127-Chol micelles diluted with phosphate buffer saline (pH 7.4).

The dilution stability of DTX-loaded optimized FA-PF127-Chol micelles was studied. The results are shown in Figure 6. It was observed that the particle size and PDI of nanomicelles remained approximately constant with no significant change after dilution for 24 h, thereby indicating the kinetic stability of micelles resulting from the hydrophobic interaction between cholesterol molecules, cholesterol molecules and PPO segments of the Synperonic molecules, and also cholesterol molecules with PPO and DTX molecules [33].

### 3.4. *In Vitro* DTX Release

**Figure 7:**
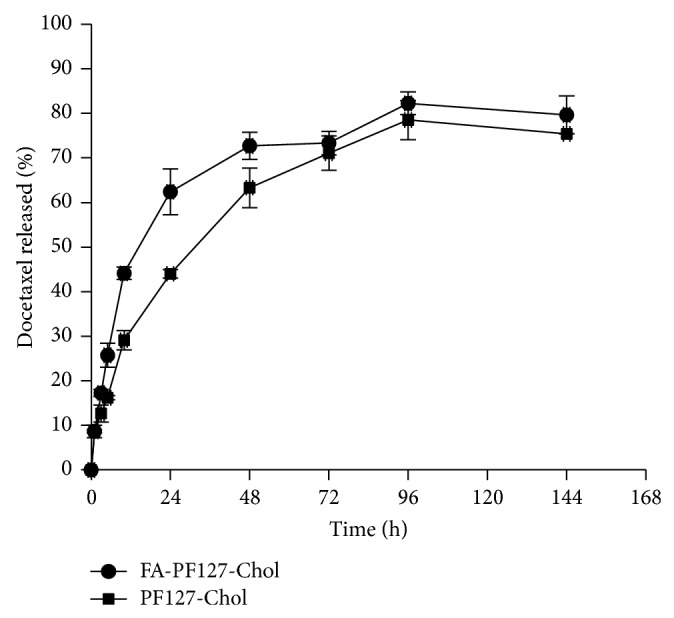
DTX release profiles from FA-PF127-Chol and PF127-Chol micelles at the pH of 7.4.

Drug release profiles from the micelles [optimized formulation of targeted micelles (formulation of P12S25) and nontargeted micelles at the pH of 7.4] are shown in Figure 7. The results showed a burst release in the first 10 h with a slower and steady release during the following 134 h. The burst release was related to the portion of DTX located on or near the surface. As release medium penetrated the tortuous path on the surface of the micelles, the drug located on the surface and near the surface was dissolved rapidly and diffused out from the micelles. The following slower and steady release was related to drug diffusion from the core of the micelles. As shown in Figure 7, the general release profile of the drug from nontargeted micelles was similar to that of the targeted ones except for a little slower release of the drug from the former. The faster release from FA-PF127-Chol micelles could be attributed to their smaller particle size, resulting in a higher contacting surface area with release media compared to PF127-Chol micelles [51].

**Figure 8:**
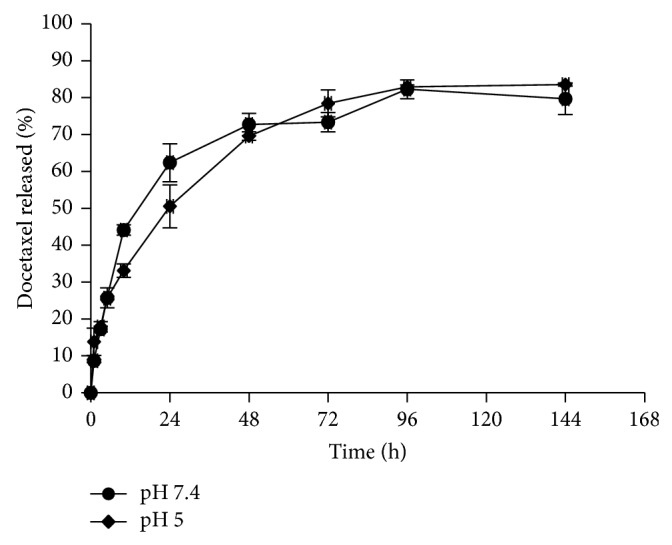
DTX release profiles from FA-PF127-Chol at different pH values.

To show the effect of pH on the release profile of DTX from FA-PF127-Chol, drug release was evaluated at two pH values. As shown in Figure 8, there was no significant difference in the cumulative amount of drug released at pH values 5 and 7.4. This finding can be attributed to the nonionic nature of the copolymer. The copolymer was stable with pH changes and its solubility was not affected by pH changes [52].

### 3.5. *In Vitro *Cellular Cytotoxicity Assay

**Figure 9:**
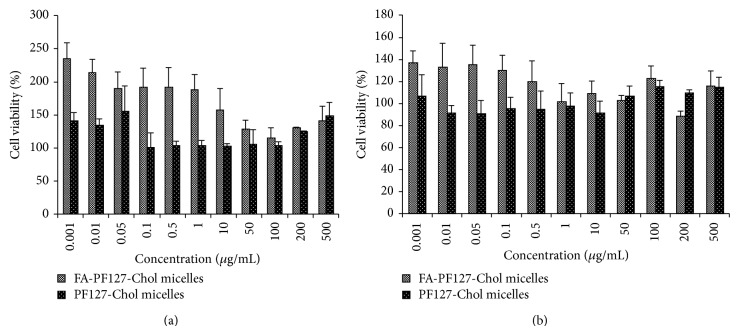
*In vitro* cytotoxicity of blank FA-PF127-Chol and PF127-Chol micelles on L929 cells after (a) 24 h and (b) 48 h incubation time.

The cytotoxicity of empty FA-PF127-Chol and PF127-Chol micelles was evaluated on L292 cells as normal cells. Results were shown in Figure 9. The nanoparticles were found nontoxic for normal cells over a range of concentration from 0.001 to 500 *μ*g/mL after 24 and 48 h of incubation with cells. So these micelles are nontoxic and are expected to be safe for biomedical application.

In order to investigate the cytotoxic potential of DTX-loaded FA-targeted micelles when compared to the nontargeted ones and the free drug, two cancerous cell lines, B16F10 melanoma cells (as FA positive receptor) and HepG2 cells (as FA negative receptor), were selected. HepG2 cell line as a FA negative cell line was used to show the role of FA in enhancing the cellular uptake.

**Figure 10:**
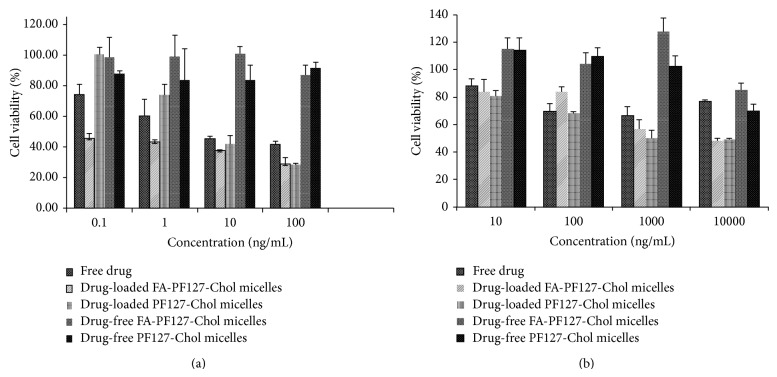
*In vitro* cytotoxicity of DTX-loaded FA-PF127-Chol micelles, DTX-loaded PF127-Chol micelles, free DTX, DTX-free FA-PF127-Chol micelles, and DTX-free PF127-Chol micelles on (a) B16F10 cells and (b) HepG2 cells.

Figures 10(a) and 10(b) show the B16F10 and HepG2 cell viability after drug exposure for 48 h, respectively. Free DTX was dissolved in water containing 1% DMSO as the cosolvent. The final concentration of DMSO in culture media was 0.1%, with no toxic effect on the cell lines. Blank (drug-free) FA-targeted micelles and the nontargeted ones (blank micelles) were also tested in equal concentrations as drug-loaded micelles on B16F10 and HepG2 cells. As shown in Figure 10, drug-free targeted and nontargeted micelles did not show significant toxicity. Zhang et al. [51] also reported no cytotoxicity for Synperonic P105 on A-cells 2780 ovarian carcinoma during 48 h incubation in spite of its potential for cell proliferation inhibition. As shown in Figure 10, cell viability of various treatments was dose-dependent and it was increased with concentration enhancement. As shown, HepG2 cell line was less sensitive to DTX compared to B16F10 melanoma cells at the same concentration. The possible reason for this finding could be attributed to the potential of hepatocyte cell to metabolize DTX. Zhang et al. [53] have shown the lower cytotoxicity of paclitaxel (PTX) on HepG2 cell line when compared to EMT6 mammary carcinoma cells due to the ability of liver cells to metabolize and detoxify PTX. As shown in Figure 10(a), FA-targeted micelles showed higher cytotoxicity, compared to free drug and nontargeted micelles, against B16F10 melanoma cells, especially in lower concentrations. At the concentrations of 0.1 and 1 ng/mL, the cell viability of FA-targeted micelles was 45.55 ± 3.21% and 43.40 ± 1.19%, respectively, which was significantly (*P* < 0.05) lower than 100 ± 4.6% and 73.68 ± 7.19% for nontargeted micelles and 73.99 ± 6.71% and 60.35 ± 10.80% for the free drug, respectively. Also, the IC_50_ value of FA-targeted micelles was about 0.1 ng/mL, which was significantly (*P* < 0.05) lower than 8.56 ± 0.596 ng/mL for the nontargeted ones and 7.97 ± 1.84 ng/mL for free DTX, respectively. At the concentration of 100 ng/mL, the drug concentration was several times more than IC_50_. Therefore, the cell viability was decreased in both targeted and nontargeted groups with no significant difference (*P* > 0.05).

Nontargeted micelles showed less toxicity compared to free DTX at the concentrations of 0.1 and 10 ng/mL. This could be attributed to the slow release behavior of the drug from the micelles which produced lower concentrations of the drug at same period of time in the cell culture medium and more rapid uptake of free drug compared to drug-loaded nontargeted micelles [11, 54]. In contrast, DTX-loaded FA-targeted micelles did not show any superior cytotoxic activity compared to nontargeted micelles in HepG2 cells (FR negative) (Figure 10(b)). A similar result was also reported for targeted folate-conjugated hyaluronic acid polymeric micelles used for paclitaxel delivery compared to the nontargeted micelles [55]. From these results, it may be concluded that more cytotoxicity of FA-targeted micelles than nontargeted ones in B16F10 melanoma cells could be explained by their enhanced cellular uptake via FR-mediated endocytosis.

### 3.6. Cellular Uptake Study

**Figure 11:**
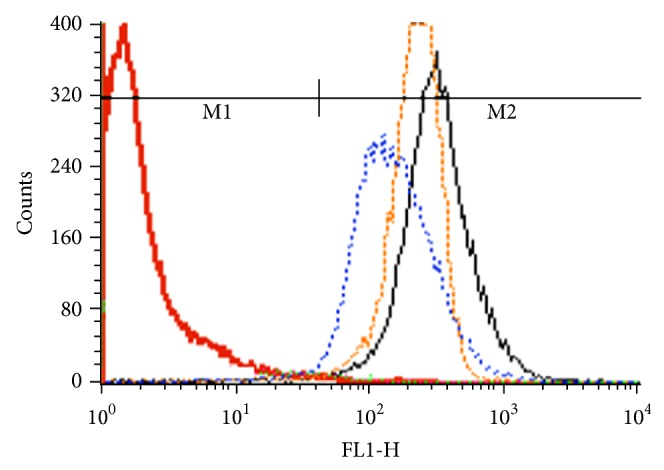
Flow cytometry profiles of B16F10 cell line after 3 h incubation with free C6 (orange), culture media (red), C6-loaded PF127-Chol micelle (blue), and C6-loaded FA-PF127-Chol micelles (black).

In this study, C6 was used as a model hydrophobic fluorescent marker for the cellular uptake study due to several advantages reported in the literature. These include biocompatibility, high fluorescence intensity, high hydrophobicity, and low leakage rate. In addition, C6 was considered as a hydrophobic drug that could not be released during the incubation period of the micelles in the studied cells [56, 57]. Flow cytometry results in Figure 11 showed more cellular uptake of C6-loaded FA-targeted micelles compared to the nontargeted ones. Based on the geometric means of each histogram in Figure 11, the mean cellular fluorescence intensity for FA-targeted micelles was approximately 2.16 and 1.35 times higher than those nontargeted micelles and free C6, respectively. This could be explained by the enhanced cellular uptake of FA-targeted micelles via FA receptor mediated endocytosis in B16F10 cells.

**Figure 12:**
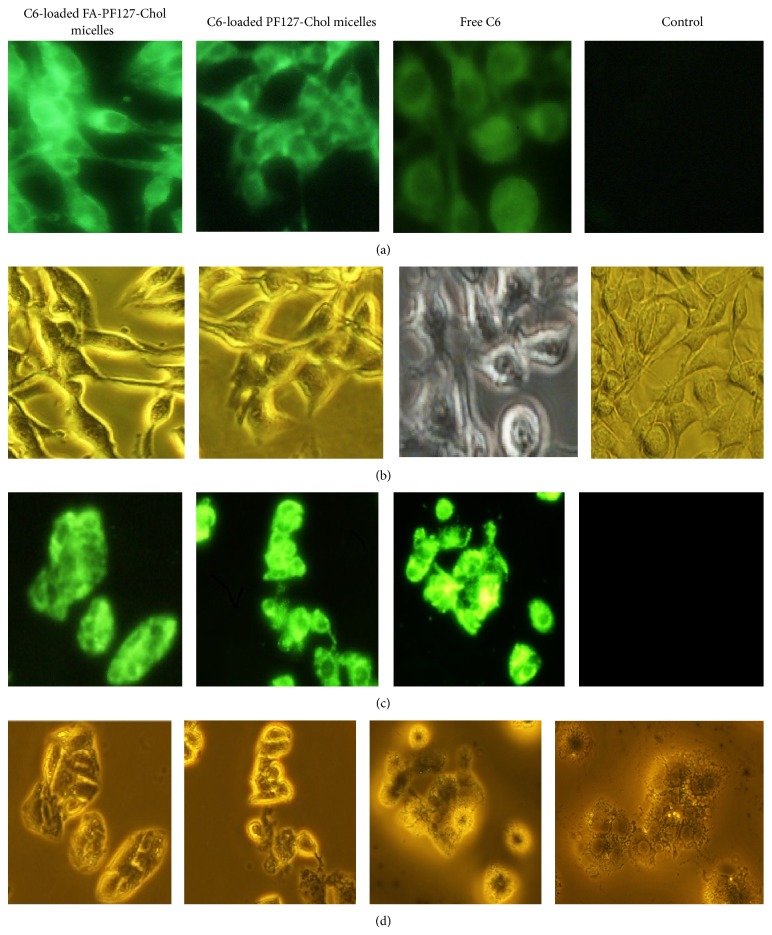
The fluorescent and visible light microscopy images of B16F10 ((a) and (b)) and HepG2 ((c) and (d)) when incubated with C6-loaded FA-PF127-Chol micelles, C6-loaded PF127-Chol micelles, free C6, and control (culture media) for 3 h at 37°C.

The florescent microscope images of B16F10 cells and HepG2 cells after 3 h incubation with different treatments of C6 are shown in Figure 12. For C6-loaded FA-targeted micelles, the images showed more fluorescence intensity compared to the nontargeted ones and free C6 in B16F10 cells. However, in HepG2 cells, which were the folate negative receptor, the fluorescence intensity in cells did not show any distinct difference between the FA-targeted micelles and the nontargeted ones.

These experiments confirmed that FA on the surface of micelles could be recognized by cell receptor and the increased cellular uptake via endocytosis facilitated the entry of fluorescence dye into B16F10 cells.

### 3.7. The* In Vivo *Antitumor Efficacy

**Figure 13:**
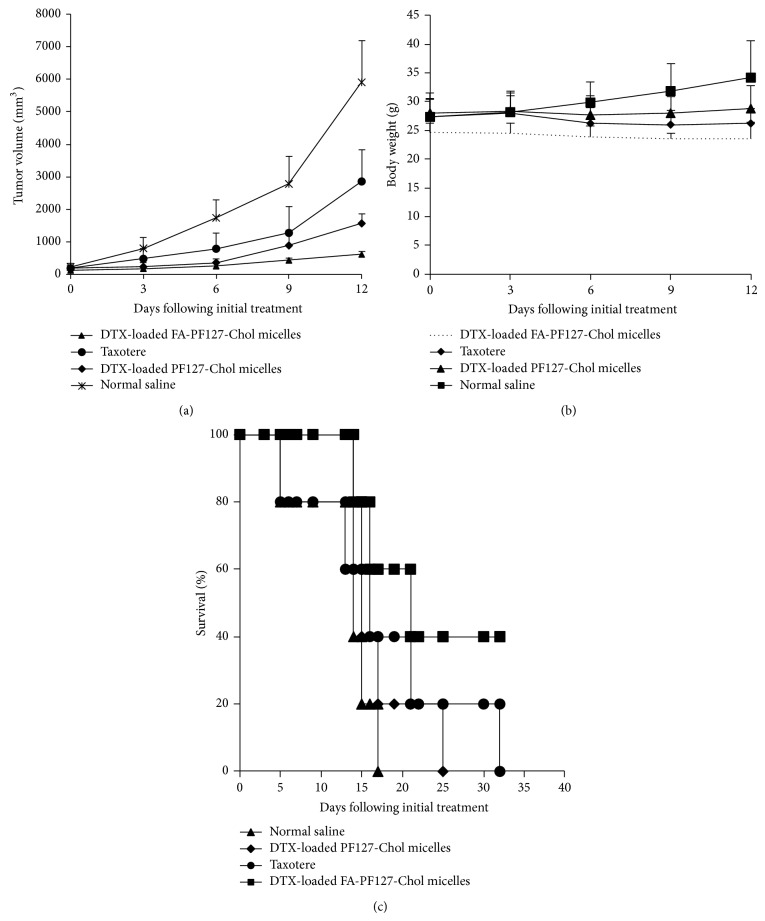
Antitumor effect of different treatment on B16F10 tumor bearing C57BL6 mice. (a) Tumor growth curves. (b) Changes in mice body weight. (c) Kaplan-Meier survival curves of tumor bearing mice.

**Figure 14:**
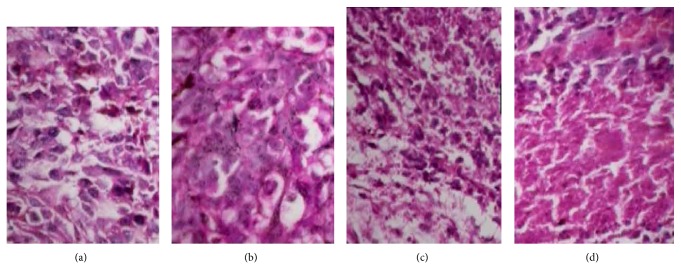
Histopathological analysis of the tumor tissue of mice treated with different formulations by HE staining. (a) Normal saline. (b) Taxotere. (c) DTX-loaded PF127-Chol micelles. (d) DTX-loaded FA-PF127-Chol micelles.

The* in vivo* antitumor efficacy of DTX-loaded micelles with or without FA and Taxotere was evaluated in B16F10 melanoma bearing mice. Taxotere is a marketed parenteral formulation of DTX based on Tween 80. DTX was formulated as Taxotere in a solution of Tween 80/ethanol/saline (20 : 13 : 67) and used as control [58]. Tumor growth curve is shown in Figure 13(a). As shown, the groups treated with DTX-loaded micelles and Taxotere inhibited tumor growth significantly in comparison with normal saline groups; at the end of experiment on day 12, tumor volume of folic acid-targeted micelles was significantly smaller (*P* < 0.05) compared with that in other treated groups. The weights of excised tumor in different treatment groups were shown in Table 5. The final tumor weight in DTX-loaded FA-PF127-Chol treated group was 0.78 ± 0.19 which was remarkably smaller than that of other treatment groups indicating DTX-loaded FA-PF127-Chol inhibited tumor growth more effectively as compared with other treatment groups. The body weight of mice was monitored as an index of systemic toxicity. Body weight of mice injected with saline gradually increased due to rapid growth of tumor. In Taxotere treated group, mice lost little body weight during study because it might be its toxic effect on normal tissue. On the contrary, DTX-loaded micelles did not change body weight of mice (Figure 13(b)). Histological analysis of tumor in saline-treated groups showed a large number of pleomorphic cells with enlarged, hyperchromatic nuclei and little cytoplasm. Different from the control group, necrosis occurred in tumor tissues of mice treated with various formulation of DTX. In particular more necrosis area and less number of tumor cells were seen in tumor tissue of mice treated with DTX-loaded FA-PF127-Chol micelles than in those treated with Taxotere and DTX-loaded PF127-Chol micelles, indicating targeted nanoparticles more effectively accumulate in tumor tissue and kill cancer cells (Figure 14). In a separate study, the survival of mice bearing melanoma tumors was evaluated to determine the antitumor efficacy.  Figure 13(c) shows the survival plot of mice bearing tumor. The means for survival time were shown in Table 5. Treatment with Taxotere and DTX-loaded PF127-Chol prolonged the mean survival time as compared with normal saline group. But the mean survival time of mice treated with DTX-loaded FA-PF127-Chol micelles exceeded and was longer than that of mice treated with DTX-loaded PF127-Chol micelles and Taxotere, indicating the survival rate of mice is more effectively improved when administered in targeted nanoparticle formulation. These findings indicated the stronger anticancer effect of DTX-loaded targeted micelles on B16F10 tumor model, contributing to more effective transport of DTX to the tumor site. Active targeting via specific ligands could improve the cytotoxicity and antitumor efficacy of drug-loaded nanoparticles. These results were in agreement with those reported by previous published articles. Gupta et al. [59] showed 5-FU-loaded FA-targeted liposome significantly inhibited tumor growth and increased survival in mice bearing tumor as compared to free 5-FU and 5-FU loaded in nontargeted liposomes. In another study, Yu et al. showed that RGD-modified sterically stabilized liposomes (SSL) incorporating doxorubicin improved anticancer activity of DOX and animal survival compared with DOX-loaded SSL in C57BL6 mice bearing melanoma [60]. These results showed encapsulation of DTX in FA-PF127-Chol micelles increases antitumor activity of chemotherapeutic drug.

## 4. Conclusion

In the present study, FA-targeted micelles of PF127-Chol were synthesized successfully for the delivery of DTX. DTX-loaded optimized FA-PF127-Chol micelles were spherical with a mean particle size of 171.3 nm, the narrow size distribution of 0.325, and the high encapsulation efficiency of 99.6%. DTX-loaded micelles showed a sustained release behaviour during 144 h. MTT assay, flow cytometry studies, and fluorescent microscopy images indicated superior cytotoxicity and more cellular uptake of FA-modified micelles compared to the nontargeted ones and free drug* in vitro*. Also, the IC_50_ value of FA-targeted micelles was about 0.1 ng/mL, which was significantly (*P* < 0.05) lower than 8.56 ± 0.596 ng/mL for the nontargeted ones and 7.97 ± 1.84 ng/mL for free DTX, respectively. The* in vivo* antitumor effects of the FA-targeted micelles showed a significant decrease (*P* < 0.05) in tumor volume within 12 days compared to free drug or drug loaded in the nontargeted micelles. DTX-loaded FA-PF127-Chol micelles showed the superior anticancer activity toward mice bearing melanoma compared with Taxotere and DTX-loaded PF127-Chol micelles. These results suggested that FA-PF127-Chol had the potential as a novel carrier for the targeted delivery of DTX in positive folate receptor cancerous cells such as melanoma.

## Figures and Tables

**Table 1:** Studied formulations of folic acid conjugated cholesterol-pluronic F127 copolymeric micelles.

Formulation code	Polymer/drug ratio	Solvent type	Dialysis temperature
P3S25	3	DMSO	25°C
P12S25	12	DMSO	25°C
P3F25	3	DMF	25°C
P12F25	12	DMF	25°C
P3S40	3	DMSO	40°C
P12S40	12	DMSO	40°C
P3F40	3	DMF	40°C
P12F40	12	DMF	40°C

**Table 2:** Physicochemical properties of drug-free FA-targeted PF127-Chol micelles and nontargeted ones.

Formulations	Particle size	Zeta potential	PDI
	(nm)	(mV)	
P3S25	99.9 ± 13.66	−8.26	0.59 ± 0.08
P12S25	144.56 ± 8.11	−4.160	0.39 ± 0.043
P3F25	139.15 ± 3.18	−2.3	0.49 ± 0.017
P12F25	91.73 ± 5.47	−3.68	0.529 ± 0.04
P3S40	113.4 ± 3.92	−1.73	0.459 ± 0.07
P12S40	86.25 ± 3.97	−2.157	0.53 ± 0.02
P3F40	192.3 ± 2.82	−4.14	0.49 ± 0.20
P12F40	131.7 ± 4.97	−3.01	0.42 ± 0.060
Nontargeted	96.53 ± 4.6	−0.952	0.542 ± 0.01

**Table 3:** Physicochemical properties of DTX-loaded FA-targeted PF127-Chol micelles and nontargeted ones.

Formulations	Loading efficiency (%)	Particle size (nm)	Zeta potential (mV)	PDI	Release efficiency (RE_144_%)
P3S25	65.39 ± 1.05	186.93 ± 17.70	−15.60	0.36 ± 0.05	64 ± 1
P12S25	99.59 ± 2.79	171.3 ± 6.42	−7.80	0.32 ± 0.07	70 ± 2
P3F25	103.18 ± 0.06	260.57 ± 62.97	−8.50	0.43 ± 0.24	40 ± 1
P12F25	52.33 ± 5.74	83.46 ± 13.30	−7.60	0.78 ± 0.21	67 ± 2
P3S40	100.04 ± 2.48	84.04 ± 6.04	−5.85	0.52 ± 0.09	78 ± 1
P12S40	100.07 ± 3.20	89.26 ± 10.63	−5.13	0.59 ± 0.05	80 ± 4
P3F40	83.45 ± 0.70	213.23 ± 6.98	−4.72	0.38 ± 0.04	50 ± 1
P12F40	72.17 ± 1.14	140.80 ± 3.70	−4.81	0.24 ± 0.03	46 ± 4
Nontargeted micelles	81.00 ± 3.00	192.00 ± 12.60	−2.12	0.20 ± 0.01	62 ± 1

**Table 4:** Physicochemical properties of DTX-loaded targeted micelles 2 months after preparation.

Formulation code	Size	Zeta potential	PDI
P3S25	171.3 ± 7.8	−14.16	0.363 ± 0.029
P12S25	156.9 ± 6.15	−10.6	0.385 ± 0.015
P3F25	268.77 ± 44.48	−8.9	0.47 ± 0.17
P12F25	137.76 ± 3.81	−9.66	0.317 ± 0.006
P3S40	137.6 ± 7.75	−9.69	0.19 ± 0.015
P12S40	156.4 ± 4.62	−10.58	0.27 ± 0.005
P3F40	207.23 ± 13.8	−7.8	0.22 ± 0.108
P12F40	157.33 ± 3.69	−5.78	0.328 ± 0.054

**Table 5:** Effect of different treatment on tumor weight and means for survival time.

Treatment groups	Tumor weight (g)	Means for survival time
Normal saline	10.92 ± 4.06	13 ± 2.074
DTX-loaded FA-PF127-Chol micelles	0.78 ± 0.19	>17.4
DTX-loaded PF127-Chol micelles	2.21 ± 0.38	17.2 ± 2.01
Taxotere	2.85 ± 1.45	17.4 ± 4.47
